# Ultra-Processed Food and Frailty: Evidence from a Prospective Cohort Study and Implications for Future Research

**DOI:** 10.3390/nu17162631

**Published:** 2025-08-14

**Authors:** Elsa M. Konieczynski, Shivani Sahni, Paul F. Jacques, Elena N. Naumova

**Affiliations:** 1 Friedman School of Nutrition Science and Policy, Tufts University, Boston, MA 02111, USA; 2 Marcus Institute, Hebrew SeniorLife and Harvard Medical School, Boston, MA 02131, USA; 3 Jean Mayer USDA Human Nutrition Research on Aging, Tufts University, Boston, MA 02111, USA

**Keywords:** nutrition, epidemiology, aging, UPFs, frailty

## Abstract

**Background:** Ultra-Processed Foods (UPFs) make up a growing share of older adults’ diets and may contribute to frailty through pro-inflammatory pathways. The objective of this study was to examine the association of UPF intake with frailty development and with annual changes in select frailty components. **Methods:** This prospective cohort study used data from 2547 participants in the Framingham Offspring Cohort. UPF intake was assessed using a food frequency questionnaire and classified according to the NOVA framework, and frailty was defined by the Fried frailty phenotype. We used cumulative and mixed logistic regression models to examine the association between daily servings of UPF and odds of developing frailty, adjusting for baseline age, education, energy intake, multivitamin use, smoking, self-rated health, history of diabetes, cancer, cardiovascular disease, and diet quality. For the frailty component analysis, we used cumulative linear regression models to assess the association between UPF intake and annual changes in grip strength, gait speed, and weight, further adjusting for BMI and physical activity. We also evaluated potential effect modification by sex and baseline age (<60 vs. ≥60 years). **Results:** The study population was 55.1% female, with a mean age of 60.3 ± 8.9 years. Over an average follow-up of 10.8 years, 233 participants (9.2%) developed frailty. UPF intake was not associated with frailty development in either the cumulative or mixed regression models. UPF intake was not associated with annual weight change but was inversely associated with annual change in gait speed and with annual change in grip strength in men only. **Conclusions:** Our findings contribute preliminary evidence that, in middle-aged and older adults, increased UPF intake is not associated with frailty but may be related to worsening muscle strength and function. Further research with a more granular approach to UPF classification is required to translate these findings to practical recommendations and to clarify their clinical significance.

## 1. Introduction

The global population is aging rapidly due to decreasing birth rates and increasing life expectancies. By 2050, it is expected that adults over 60 years of age will outnumber youth and young adults aged 10–24 years [1]. In this context, age-related diseases such as frailty have emerged as a major public health concern.

Frailty is a multisystem condition characterized by decreased physiological reserve and increased vulnerability to stressors [2,3]. It is associated with increased risk of disability, hospitalization, morbidity, and mortality [4,5,6]. Frailty is commonly defined using the Fried frailty phenotype, which classifies individuals as frail if they exhibit three or more of the following: unintentional weight loss, weakness, exhaustion, slowness, and low physical activity [7]. In the U.S, approximately 15% of adults over 65 years of age experience frailty [8]. Twin studies estimate that genetic factors account for 25–45% of the variability in frailty, with the remainder attributed to individual-level environmental factors [9,10,11,12]. Therefore, there is great interest in identifying modifiable environmental factors, including nutrition, that influence frailty and could be leveraged as a preventative strategy against this condition.

Protein and energy undernutrition results in tissue catabolism and muscle loss [13,14], and has been long understood as a frailty risk factor [15]. Other dietary characteristics may contribute to frailty development, potentially through interaction with oxidative stress and inflammatory pathways that have been implicated in the pathogenesis of frailty. For example, higher consumption of unprocessed foods and greater adherence to dietary patterns that are associated with lower oxidative stress and inflammation, such as carotenoids [16], fruits and vegetables [17], and antioxidants [18,19], and a Mediterranean dietary pattern and the alternative Healthy Eating Index [20,21,22] are associated with a lower risk of frailty development. Conversely, higher dietary inflammatory index scores [23,24] are associated with a higher risk of frailty.

Ultra-Processed Foods (UPFs) make up a growing portion of the older adult diet [25] and may be relevant to frailty [26]. UPFs are defined according to the NOVA framework as “formulations of ingredients, mostly for industrial use only, derived from a series of industrial processes” [27]. UPFs tend to be high in added sugars, sodium, saturated and trans fats, and energy while providing low amounts of fiber, protein, and essential micronutrients necessary for a balanced diet and healthy aging [28]. These characteristics, together with the addition of cosmetic additives, make UPFs hyperpalatable and appealing [29]. The poor nutritional quality, high energy density, and palatability, among other characteristics, make UPFs a plausible pro-inflammatory factor and may link UPF intake to musculoskeletal decline and frailty development [26].

There is limited research on the relationship between UPF intake and frailty. Two cross-sectional studies reported that higher UPF intake is associated with increased odds of frailty [30,31]. To the best of our knowledge, only two prospective cohort studies have examined this relationship. One study followed 1822 men and women in the Seniors-ENRICA cohort over 3.5 years and defined frailty using the Fried phenotype [32], while the other analyzed data from 63,743 women in the Nurses’ Health Study for up to 26 years and defined frailty using the FRAIL scale [33], which is moderately correlated with the Fried phenotype [34]. Both studies reported that higher UPF intake was associated with a greater risk of frailty. Further longitudinal investigation of the relationship between UPF consumption and frailty, particularly with substantial follow-up in both men and women and using the validated Fried frailty phenotype, will contribute valuable further evidence on this topic.

This study aimed to examine the relationship of UPF consumption with odds of frailty development and with annual changes in related frailty phenotype components of grip strength, gait speed, and weight change in middle-aged and older adults from the Framingham Heart Study (FHS) Offspring cohort. We hypothesized that daily UPF intake would be associated with greater odds of frailty development and decreased grip strength and gait speed. Based on prior literature linking UPF intake with weight gain [35,36,37,38], we anticipated that UPF intake may be associated with increased weight over follow-up. We also discussed the limitations and challenges of the current UPF classification and offered pathways to enhance our understanding of how UPFs affect health.

## 2. Materials and Methods

### 2.1. Study Population

This prospective cohort study used data from the Framingham Heart Study (FHS) Offspring cohort. The FHS began in Framingham, Massachusetts, in 1948. It originally enrolled 5209 adults aged 28–62 years of age. Participants returned biennially for data collection, including physical examinations, laboratory tests, cardiovascular disease (CVD) assessments, and questionnaires. In 1971, 5124 offspring of the original cohort participants were enrolled in the FHS Offspring cohort, undergoing similar assessments approximately every four years. Dietary assessment was added to the fifth examination cycle in 1991–1995. We used data from the seventh (1998–2001, *n* = 3539), eighth (2005–2008, *n* = 3021), and ninth (2011–2014, *n* = 2430) study examinations for the primary analysis and included data from the fifth (1991–1995, *n* = 3799) and sixth (1995–1998, *n* = 3532) examination cycles for sensitivity analyses. The original FHS data collection protocol and procedures were approved by the Institutional Review Board of Boston University Medical Center. The present study was approved by the Institutional Review Board of Tufts University (STUDY00004638).

**Figure 1:**
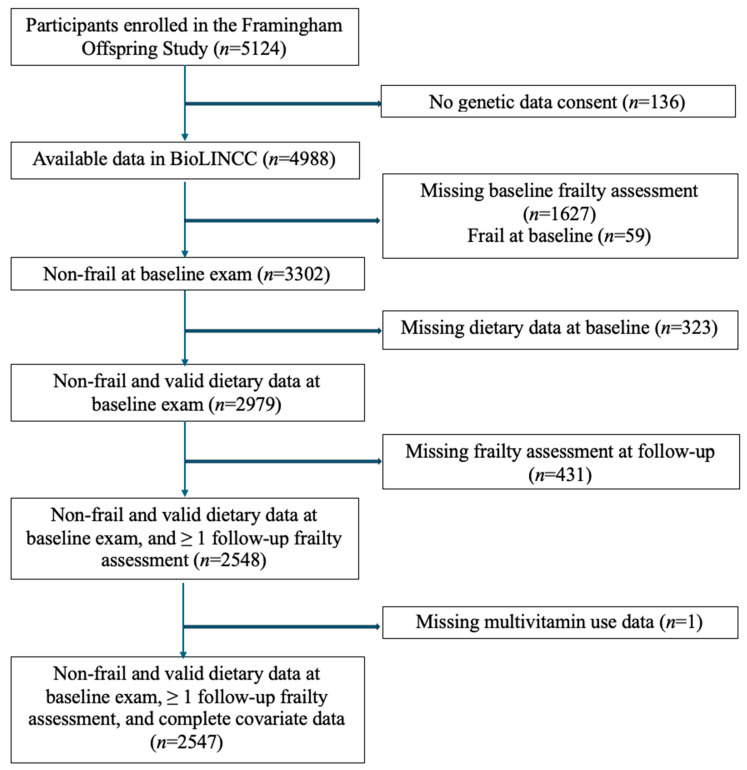
Flowchart of participants in study.

Our sample included all participants who had diet and frailty data at our baseline assessments, were not frail at baseline, had a follow-up frailty assessment, and had information on relevant covariates. Figure 1 describes the flow of participants into the analytic sample. The data were sourced from BioLINCC, which included only participants who consented to genetic data sharing (*n* = 4988). We excluded 1627 participants who were missing a baseline frailty assessment (i.e., no frailty assessment at examinations seven or eight) and 59 participants who were frail at baseline. Among the remaining 3302 non-frail participants, we excluded 323 individuals missing dietary data at baseline and then 431 who lacked a follow-up frailty assessment. Finally, one participant was excluded due to missing non-categorical covariate data across all three examination cycles (missing data for covariates treated exclusively as categorical throughout the analyses were coded into a separate ‘missing’ category). The final sample for the frailty analysis included 2547 participants.

### 2.2. Diet Assessment

The usual diet over the past year was assessed using the Harvard 126-item semiquantitative food frequency questionnaire (FFQ) [39]. The FFQ provides a list of foods with standard serving sizes, and participants report their frequency of consumption using nine categories, ranging from “never or less than once per month” to “6+ times per day.” Participants could also list up to three additional foods not included in the standard 126-item list, along with their frequency of consumption.

FFQs were considered invalid if more than 12 items were left blank, or if total energy intake was <600 kcal/day or >4000 kcal/day for women and <600 kcal/day or >4200 kcal/day for men. Nutrient intakes were calculated by multiplying the frequency of consumption for each food item by its nutrient content. Nutrient composition was based on the USDA food composition database and other published sources [40].

UPF intake was defined by the NOVA classification system [27] and was the exposure of interest. FFQ items were classified as UPF or non-UPF using the NOVA assignments published by Khandpur et al. [41]. This classification was developed through independent coding by three researchers, and discrepancies were resolved through consultations with experts and additional resources (research dietitians and grocery store scans). Two researchers on our team independently classified the 349 items in the free form ‘Other Foods’ section of the FFQ that were not classified in the paper by Khandpur and colleagues, and discrepancies between reviewers were decided by consensus. Daily servings of UPF were energy-adjusted using the residual method and centered at 2000 kcal [42] and evaluated as a continuous variable.

### 2.3. Frailty Assessment

Frailty was defined using the Fried frailty phenotype, which consists of five components: unintentional weight loss, weakness, exhaustion, slowness, and low activity [7]. The phenotype was originally conceptualized by Fried et al. and evaluated using data from the Cardiovascular Health Study, with the components defined as follows: unintentional weight loss as self-reported weight loss of ≥4.5 kg or ≥5% of body weight in the past year; exhaustion as a response of “occasionally a moderate amount of time” or “most of the time” to either of the Center for Epidemiologic Studies Depression Scale (CES-D) questions, “I could not get going” or “I felt that everything I did was an effort”; weakness as grip strength (kg) in the lowest sex- and BMI-specific quintile; slowness as a 15 ft gait speed (m/s) in the lowest sex- and height-specific quintile; and low physical activity as energy expenditure in the lowest sex-specific quintile (estimated in kilocalories). Individuals were classified as robust if they met none of the components, pre-frail if they met one or two components, and frail if they met three or more components [7].

Based on prior work, we adapted some of the Fried frailty criteria according to the data available in the Framingham Offspring Study [43,44,45,46]. Low activity was assessed using the Physical Activity Index (PAI) [47], reflecting the sum of sedentary, slight, moderate, and vigorous activity as metabolic equivalent of task (MET-hrs/d). We determined the cut-off threshold for the lowest quintile of the PAI score at the baseline examination and held this threshold constant in subsequent exams to identify participants meeting the criterion for low physical activity. Also consistent with prior work [43,44,45,46], weight loss was defined as losing more than 10 lbs. in the past year, having an underweight BMI (<18.5 kg/m^2^), or experiencing an annualized weight loss of more than 10 lbs. per year. Additionally, participants who met the weight loss criterion at any given examination were assumed to continue meeting this criterion, as it is unlikely for individuals to sustain a yearly loss of 10 lbs over time. Exhaustion was defined according to Fried’s original criteria, using responses from the CES-D.

Weakness and slowness were also defined according to the grip strength and gait speed cut-offs established by Fried et al. [7]. Grip strength was assessed on a Jamar Hydraulic Hand Dynamometer (Lafayette Instrument, Lafayette, IN, USA) and recorded in kg of force. Participants were seated in a chair with their dominant-side forearm supported on the arm of the chair, and with their elbow flexed to a 90-degree angle. Participants were then instructed to squeeze the dynamometer for 3 s with their maximum strength. Three trials were attempted on each hand for a total of six trials. We selected the highest value across the six trials (regardless of the side, i.e., left vs. right) for analysis.

Gait speed was assessed via a 3 or 4 m walk test. Participants were instructed to walk the distance at their normal pace. Gait speed was derived by dividing the walk time by the length of the course. Consistent with prior studies, individuals who reported a functional limitation as the reason for missing grip strength or gait speed measurements were coded as meeting the weakness and/or slowness criteria [43].

We classified individuals as frail if they met three or more criteria, and as non-frail if they met two or fewer criteria. Individuals were not classified if they did not have sufficient component data to definitively determine their status. For example, to be considered non-frail, participants needed to complete assessments for three criteria that they did not meet.

### 2.4. Covariates

To minimize potential confounding, several demographic and lifestyle factors related to frailty were considered as covariates. These included sex, age, and the highest level of education achieved as a proxy for socioeconomic status (categorized as did not graduate from high school, high school graduate, some college, college graduate, and missing). Current smoking status was recorded as “yes” or “no” for regular smoking in the past year. Self-reported health status was categorized into three groups: Fair/Poor, Good/Very Good, and Excellent. Physical activity was characterized by the PAI described above.

Diet-related covariates included multivitamin use (yes/no) and daily energy intake (kcal/day), which were collected via the FFQ. Diet quality was also assessed using a modified dietary approaches to stop hypertension (DASH) diet score, which accounted for intake of fruits, vegetables, sugar-sweetened beverages, nuts and legumes, red meat, milk, whole grains, and sodium, based on prior work [48,49]. Briefly, we calculated the energy-adjusted residual of each of these components and categorized the residual in quintiles. A value of 1 was assigned to the lowest quintile up to a value of 5 for the highest quintile, except for the components of red meat, sugar-sweetened beverage, and sodium, in which the scoring was reversed. The DASH score was the sum of the component scores, ranging from 8 to 40, in which a higher score indicates better diet quality [48,49].

Indicator variables were created for the following conditions: history of type 1 or type 2 diabetes (defined as oral glucose tolerance test plasma glucose ≥ 200 mg/dL, or ≥126 mg/dL fasting blood glucose, or use of diabetes medication, at a past examination), history of non-skin cancer (validated by health record review), and history of CVD, which included coronary heart disease, congestive heart failure, stroke, transient ischemic attack, or intermittent claudication.

Participants’ weight and height were measured during the examinations. Body mass index (BMI) was calculated by dividing weight (in kg) by height (in m^2^). To maximize the available frailty assessments, missing weight and height data were imputed by carrying the last observation forward. If BMI was missing, it was calculated using available or imputed weight and height. If weight or height measurements were unavailable, the last non-missing BMI value was carried forward.

### 2.5. Statistical Analysis

The baseline characteristics of the study population were summarized as mean (SD) for continuous variables and percentages for categorical variables. We compared the baseline characteristics of participants who were included in the study and those who were excluded due to missing follow-up data to investigate potential loss to follow-up bias. We also compared the baseline characteristics of participants across high vs. low UPF intake, using the median intake as the cutoff. Additionally, we assessed the correlation between energy-adjusted servings of UPFs and DASH score.

We used cumulative and mixed effects logistic regression models to examine the relationship between UPF intake and frailty. The two different approaches allowed us to evaluate frailty as a terminal condition (cumulative model) and a dynamic condition (mixed model), and to model the chronic (cumulative model) and immediate effects (mixed model) of the independent variables.

In the cumulative regression models, participants were followed from their baseline assessment until their censoring visit, defined as either their first frailty diagnosis or their last assessment with available frailty data, whichever occurred first. UPF intake was updated during follow-up when additional data were available. For instance, events at the ninth examination were linked to the average UPF intake from the seventh, eighth, and ninth examinations if the baseline was the seventh examination, or from the eighth and ninth examinations if the baseline was the eighth examination. Covariates such as multivitamin use, current smoking status, energy intake, alcohol consumption, DASH score, and self-rated health were updated similarly. History of CVD, cancer, or diabetes was assessed at the censoring visit. Age, education, and sex were treated as fixed variables, based on baseline data.

The cumulative logistic regression models were used to estimate the odds ratio (OR) and 95% confidence intervals of frailty development for each one-serving increase in UPF intake. Model 1 was adjusted for baseline age, education, and sex. Model 2 was additionally adjusted for energy intake, multivitamin use, smoking status, self-rated health, history of diabetes, history of cancer, and history of CVD. Model 3 was further adjusted for the DASH score.

For the mixed-effects approach, participants were not censored after frailty onset. UPF intake and covariates such as multivitamin use (categorical), current smoking (categorical), energy intake, DASH score, and self-rated health (categorical), as well as history of CVD, cancer, and diabetes, were treated as time-varying covariates and updated at each examination. Mixed-effects logistic regression models with a random intercept for the participant were used to evaluate odds ratios of developing frailty per one serving increase of UPF intake, using the same adjustment scheme specified above.

To better understand the effect of UPF intake on the frailty-related components, we conducted separate linear regression models to estimate the association between UPF intake and annualized changes in grip strength, gait speed, and weight. The annualized change was calculated by subtracting the measure at the censoring examination from the measure at the baseline examination, divided by the years between the baseline and censoring visit. The same adjustment scheme was applied, but with physical activity (all) and BMI (grip strength and gait speed only) added as additional covariates in models 2 and 3.

Because frailty is more common in women than men and in older ages compared with younger ages [8], we evaluated potential effect modification by sex and baseline age (cut off of <60 vs. ≥60 years, selected for balanced sample sizes) first by performing stratified analyses and second by comparing the fit of the model with an interaction term between UPF intake and the stratifying variable to the model without the interaction term.

We conducted several sensitivity analyses to assess the robustness of the results, including (1) calculating frailty without imputed height, weight, or BMI data, (2) considering participants reporting a functional limitation for grip or gait speed as not fulfilling the corresponding weakness or slowness criteria, and (3) using cumulative dietary intake from examination 5.

All analyses were conducted using SAS software (version 9.4; SAS Institute, Cary, NC, USA). Two-sided *p* values < 0.05 were considered statistically significant. We followed the STROBE (Strengthening the Reporting of Observational Studies in Epidemiology) recommendations for reporting of cohort studies [50].

## 3. Results

The baseline characteristics of the study population are presented in Table 1. Overall, 55.1% of participants were female, with a mean ± SD age of 60.3 ± 8.9 years, BMI of 28.1 ± 5.3 kg/m^2^, and energy-adjusted UPF intake of 7.2 ± 2.9 servings/day. Men had a higher energy-adjusted UPF intake than women (7.4 ± 3.0 vs. 7.0 ± 2.9 servings/day), and participants 60 years of age and older had a higher energy-adjusted UPF intake compared with participants less than 60 years of age (7.4 ± 2.9 vs. 6.9 ± 3.0 servings/day). Compared with the analytic sample, the 431 participants excluded due to missing follow-up frailty assessments were older (mean age 66.7 ± 8.9 years at baseline), more likely to be smokers (15.8% vs. 11.1%), and had lower grip strength (30.4 ± 11.9 vs. 33.6 ± 12.9 kg) and gait speed (1.1 ± 0.3 vs. 1.3 ± 0.3 m/s). They were also more likely to have a history of CVD (23.7% vs. 10.1%), cancer (16.0% vs. 8.3%), and diabetes (20.7% vs. 10.3%).

The baseline characteristics of participants by high (≥6.7 servings/day) and low (<6.7 servings/day) UPF intake are shown in Supplementary Table S1. Compared to those with low UPF intake, participants with high UPF intake had a slightly higher BMI (28.5 ± 5.4 vs. 27.7 ± 5.2 kg/m^2^) and a higher prevalence of CVD (11.8% vs. 8.4%), cancer (8.9% vs. 7.8%), and diabetes (12.2% vs. 8.3%) history. They were also more likely to report fair/poor health status (6.1% vs. 3.6%) and to have a high school degree or less as their highest level of education (35.1% vs. 27.4%). PAI was similar between the high and low UPF intake groups (37.6 ± 6.1 vs. 38.2 ± 6.3, respectively).

### 3.1. Frailty Analysis

Over an average follow-up of 10.8 years, 233 (9.2%) of participants developed frailty. The prevalence of frailty was 4.0% at examination 8 and 7.8% at examination 9. There were nine participants who transitioned from frail at examination 8 to non-frail at examination 9.

We observed no significant association between UPF intake and odds of developing frailty across all levels of adjustment in both the cumulative models and the mixed models (Table 2). There was no significant interaction between UPF intake and sex or baseline age (Table 3). Notably, the DASH score was inversely associated with odds of developing frailty in both the fully adjusted cumulative model (OR: 0.94, 95% CI: 0.91–0.98, *p* = 0.002) and mixed model (OR: 0.82, 95% CI: 0.76–0.87, *p* < 0.001). Additionally, UPF intake was inversely correlated with DASH score at baseline (R = −0.27, *p* < 0.001).

### 3.2. Frality Components

The grip strength analysis included 1872 participants with grip strength data at the censoring exam and the baseline exam. There was no relationship between UPF intake and annualized change in grip strength in the overall sample (Table 4) or effect modification by age; however, there was effect modification by sex (P interaction = 0.01). In men, each additional serving of UPF intake was related to a 0.02 kg (95% CI: −0.05, −0.001, *p* = 0.04) annual decrease in grip strength in the fully adjusted model, whereas, in women, there was no relationship between UPF intake and annualized change in grip strength (Table 5).

The gait speed analysis included 2033 participants with data available at both baseline and the censoring examination. Higher UPF intake was associated with a modest but statistically significant annualized decline in gait speed across all models (Table 4). In the fully adjusted model, each additional serving of UPF was linked to a decrease of 0.001 m/s per year (95% CI: −0.001, −0.000, *p* = 0.03). We did not observe any effect modification by sex or baseline age (Table 5).

The weight change analysis included 2545 participants with data available at both baseline and the censoring examination. There was no significant association between UPF intake and annualized weight change overall (Table 4) or in the sex- and age-stratified analyses (Table 5).

The results of the first two sensitivity analyses were consistent with the primary analysis (Supplementary Table S2). In the third sensitivity analysis, in which UPF was a cumulative average from examination 5, higher cumulative UPF intake was associated with lower odds of frailty in the mixed logistic regression model (Model 3: OR = 0.62, 95% CI: 0.46–0.82, *p* < 0.01). Results in the cumulative logistic regression model were consistent with the primary analysis.

## 4. Discussion

In this analysis of 2547 adults in the Framingham Offspring Cohort, we did not observe a relationship between UPF intake and the odds of developing frailty over a mean follow-up of 10.8 years. UPF intake was inversely related to gait speed in the overall sample and was associated with a decrease in grip strength in men; however, the effect sizes were small, and the clinical significance of these findings is unknown. UPF intake, as measured in the presented analysis, was not associated with the annual change in weight.

### 4.1. Our Findings in Context

The lack of an association between UPF intake and frailty contrasts with findings from two previous longitudinal studies. Fung et al. reported that higher UPF intake, assessed via the Harvard FFQ, was associated with increased risk of frailty in 63,743 women from the Nurses’ Health Study over up to 26 years of follow-up. Frailty was measured using the FRAIL scale, based on self-reported fatigue, low strength, reduced aerobic capacity, multiple chronic illnesses, and weight loss [34]. Similarly, Sandoval-Insausti et al. found that higher UPF intake, assessed as a percentage of total energy intake using an open-form diet history questionnaire [51], was associated with greater odds of incident frailty (Fried phenotype) in 1822 adults aged 60 years or older from the Seniors-ENRICA cohort over 3.5 years of follow-up [32].

Several factors may explain these contrasting findings. First, studies on UPF intake rely on researchers’ interpretations of the NOVA classification system, leading to variability in which foods are categorized as UPFs. Prior research shows poor agreement among researchers when classifying foods, even when ingredient lists are available [52]. We used the same classification reference [41] as Fung et al. [34], but it is difficult to know how our classification compared with the Sandoval-Insausti group, given the different dietary assessment methods. Second, frailty assessment methods differed across these studies, potentially contributing to inconsistent results. Fung et al. used the self-reported FRAIL scale, which is moderately correlated with the Fried frailty phenotype (r = 0.617, *p* < 0.001) [53]. Both our study and that of Sandoval-Insausti et al. used the Fried frailty phenotype but with slight adaptations. For example, Sandoval-Insausti et al. applied weakness and slowness cut-offs specific to their sample, defined low physical activity as walking <2.5 h/week for men and <2 h/week for women, and had a different grip strength protocol (measured on the dominant hand only) [32]. In contrast, we used the original cut-offs published by Fried et al., defined low physical activity as the lowest quintile of the physical activity index, and selected the maximal grip strength across both hands [7]. Finally, the baseline age of our participants was relatively young at 60.3 years, given that frailty prevalence increases rapidly after age 75 [8]. However, no significant interactions between frailty and age were observed in our study to suggest that associations would be different across those aged <60 years versus those aged ≥60 years.

Diet quality, represented by a modified DASH score, was inversely associated with the odds of developing frailty in our study. This finding is consistent with prior research [54,55,56,57,58,59] and may reflect the protective effects of whole grains, fiber, and micronutrients [60]. UPFs are typically low in these nutrients and high in salt, sugar, and fat, and, therefore, contribute to poor diet quality [61,62,63,64,65]. For this reason, it is important to adjust for diet quality in analyses of UPF and health to distinguish the effects of processing from nutrient content. In our population, UPF intake in energy-adjusted servings/day was modestly but significantly correlated with the DASH score (r = −0.27, *p* < 0.001).

To the best of our knowledge, no other study has assessed the relationship between UPF intake and gait speed. In our study, higher UPF intake was inversely associated with gait speed, with each additional serving linked to a 0.001 m/s annual decrease in gait speed. Gait speed in older adults reflects lower body strength and aerobic capacity [66] and predicts disability [67], functional dependence [68], and mortality [69]. Inflammatory markers have been associated with both lower gait speed [70,71] and gait speed decline in older adults [72]. Since UPFs may promote a pro-inflammatory state due to their high sugar and fat content, low fiber and micronutrient levels, and additives like emulsifiers and non-nutritive sweeteners [73], this may be a mechanism linking UPF intake and gait speed.

We found that UPF intake was inversely associated with modest annual decreases in grip strength in men but not in women. This finding adds to the growing body of evidence on the relationship between UPF intake and muscle health. Zhang et al. reported that higher UPF intake was associated with an annual decrease of 0.37 kg in grip strength over a median follow-up of 3 years in 5409 adults aged 40 and older, with no evidence of an interaction by sex. Grip strength was measured by a different protocol, with participants in the standing position, making it difficult to compare the absolute effect size with our study [74]. Several studies have also reported an inverse relationship between UPF intake and lean mass, which is correlated with grip strength [75], but it is a less sensitive predictor of falls and fractures [76] and mortality [77]. This association has been observed in young and middle-aged adults, as well as adolescents [78,79,80]. Sex-specific findings were reported in a longitudinal study of 1021 young adults (aged 23–25 years), where UPF intake was associated with decreased percent lean mass in women but not in men after 13–15 years of follow-up [81].

We did not detect a significant relationship between UPF intake and annual change in weight, in contrast to several other longitudinal studies in middle-aged and older adults that report associations between UPF intake and increased weight. In the EPIC cohort (*n* = 348,748; median age approximately 51 years), higher UPF intake was associated with weight gain and increased risk of overweight and obesity over 5 years [35]. Similarly, in the NutriNet-Santé cohort (*n* = 110,260; mean baseline age 43.1 years), UPF intake was positively associated with BMI increase over 4.1 years of follow-up [36]. The SUN (University of Navarra Follow-Up) study, with 8451 middle-aged participants, reported that higher UPF intake was linked to a greater risk of developing overweight and obesity over 8.9 years [37]. In the ELSA-Brasil cohort (*n* = 11,827; mean baseline age 51.3 years), higher UPF intake was associated with increased weight and waist circumference over 3.8 years [38]. The inconsistencies in findings could be due to many factors, including conceptual and technical differences in study designs and implementations.

We utilized weight change and BMI in our analyses, in keeping with the Fried frailty phenotype; however, these metrics are not straightforward indicators of health in older adults. Lean mass loss typically begins in the third decade of life and is compensated for by fat mass increases until around age 70, when total body weight tends to decline [82]. Small decreases in weight appear to be a normal part of aging in healthy adults [83], and even larger amounts of intentional weight loss coupled with appropriate exercises can improve quality of life and physical function in overweight adults while mitigating unfavorable losses in bone and muscle catabolism [84]. On the other hand, unintentional weight loss exceeding 5% of body weight may signal serious underlying disease and is associated with unfavorable musculoskeletal catabolism, morbidity, and mortality [85] and is a strong prognostic criterion for frailty [86]. Measures such as waist circumference or fat mass measures may therefore better reflect adiposity and metabolic disorder in older adults than body size alone [87]. Future studies should consider these complementary indicators of body composition to better capture the relevant metabolic and musculoskeletal context of UPF intake and weight in older adults.

### 4.2. Strengths and Limitations

This study had several strengths. It addressed a research question on the relationship between UPF intake and frailty, which had minimal prior literature. To our knowledge, it also provided the first evidence on the relationship between UPF intake and gait speed in older adults. The follow-up period of 10 years allowed us to investigate long-term dietary exposure and to update covariates across examinations using data from a community-based cohort study. The sample size was also sufficiently large, and this study was well-powered to detect even modest associations. We evaluated the relationship between UPF intake and frailty using two modeling approaches, allowing us to account for both chronic and more immediate exposure to UPFs. Additionally, we used the Fried frailty phenotype, a highly validated measure, and applied a published UPF classification system for standardized and reproducible methods. However, this study also had limitations. The population was predominantly White (>99% of our study sample), which limits the generalizability of our findings to more diverse populations. There is also likely a loss to follow-up bias given that our included population was generally healthier compared to those excluded due to a lack of frailty follow-up assessment. Dietary intake was self-reported, making it susceptible to measurement error and recall bias. We could not characterize UPF intake as % energy from UPFs because we did not have access to the energy contributions of each FFQ item. Additionally, the observational design prevents us from drawing causal conclusions. While we adjusted for key covariates, the possibility of residual confounding remains.

### 4.3. Future Directions

Our results indicate an association between UPF intake and decreased grip strength and gait speed, but not frailty. These are hypothesis-generating findings that suggest certain types of modern food processing may negatively impact healthy muscle aging and contribute to a growing body of literature linking UPF intake with chronic disease. However, to advance this body of research toward actionable conclusions, we need to reform how food processing is evaluated. While the NOVA classification system has been instrumental in driving research on food processing and health, it also has limitations that are important to contextualize the findings of UPF research.

The NOVA classification system simplifies the complex, real-world context of nutrition, UPFs, and health, making its application and interpretation challenging [88,89]. For example, age-related factors, including gastrointestinal and hormonal changes, psychiatric conditions, medications, comorbidities, and socioeconomic barriers, can limit the desire for and the ability to access and prepare nutritious food, potentially contributing to unintentional weight loss in older adults [90]. Some ‘ultra-processing’ may help older adults overcome these challenges by enhancing food palatability, improving accessibility (less preparation required and longer shelf life), and providing a softer texture. While it would be unfavorable to recommend nutrient-poor UPFs to older adults, given the importance of diet quality in the development of age-related diseases like frailty, other nutrient-dense UPFs can contribute to meeting dietary guidelines. For example, whole grains provide fiber, micronutrients, and phytoestrogens and have been linked with improved glucose tolerance [91] and decreased abdominal fat [92] and metabolic syndrome [91] in older adults. The top contributors to whole grain intake in older adults are bread and cold breakfast cereals, both of which are classified as UPFs [91,93]. Ultimately, providing broad generalizations on the UPF group as a whole could steer people away from UPFs that contribute valuable energy and nutrients. A more granular view of the UPF category in future research is required to make evidence-based dietary recommendations that incorporate these realities.

**Figure 2:**
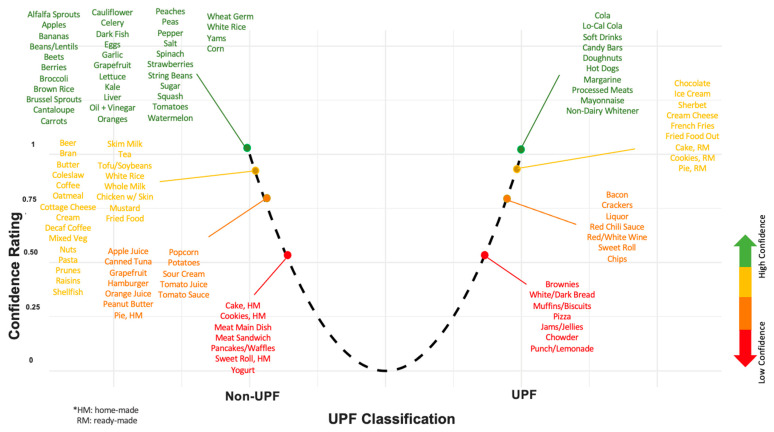
Depiction of the UPF classification of foods in the 126-item FFQ, positioned and color-coded according to our rating of confidence in the classification. The * text describes abbreviations used: home-made (HM) and ready-made (RM).

Other challenges in UPF assessment are due to the nature of the cohort study design. Cohort studies provide a valuable opportunity to examine exposures and diseases longitudinally, but have inherent financial and logistical considerations that inform data collection and methodology. For example, FFQs are widely used in prospective cohort studies because they are less resource-intensive and more cost-effective than methods such as 24-h dietary records, but they sacrifice detail, including ingredient- and product-specific information. Data collection methods also reflect research priorities at the time of study design. While existing data can be leveraged to address contemporary questions like those related to UPFs, doing so introduces potential error and reproducibility concerns. For example, the NOVA framework, proposed in 2010, postdates the Harvard FFQ and is based on ingredient contents. In applying the NOVA framework to FFQs, researchers must make subjective judgments, leading to variability in interpretation and inevitable misclassification, especially when both UPF and non-UPF versions of a food likely contribute to the same category. For example, Figure 2 shows how we rated our certainty in our UPF classification for each FFQ item. While some items like ‘Candy Bars’ and ‘Processed Meats’ obviously fulfill the UPF criteria, others, like ‘Brownies’ or ‘White/Dark Bread’, may or may not depending on their ingredient composition.

We did not have the resources for multiple expert input on this uncertainty rating, and so we did not pursue dividing the nutrient contribution of a food item to reflect uncertainty as has been done in prior work [94,95]. Initiatives to develop a UPF-specific FFQ [96] could also help to limit misclassification error. However, it is unclear if these efforts will be enough to progress UPF research towards scientifically rigorous and practically meaningful conclusions.

Classifying UPF intake as a single exposure aligns with the level of dietary detail in cohort studies, and this strategy has been useful for generating hypotheses about UPFs and health. However, this approach ultimately will not provide a way forward for scientifically rigorous or actionable guidance for consumers or food manufacturers. Grouping all UPFs together limits our ability to parse out the effects of nutritional value versus processing and to identify the mechanisms at play in the observed relationship between UPF intake and chronic disease. Furthermore, recommending complete avoidance of UPFs is impractical, as they are deeply embedded in our food system and ways of life, contributing the majority of caloric intake and grocery store offerings [97]. A more targeted approach to reformulation, or consumer education, would be more feasible and scientifically aligned, but current research lacks the granularity needed to guide such efforts. Thus, it is important for the nutrition science community to pursue research methods, including harnessing the use of Artificial Intelligence (AI) that can enhance the comprehensiveness and granularity of UPF research.

### 4.4. AI, UPF Research, and UPF Policy

AI refers to machines performing tasks that typically require human intelligence [98]. The ability of AI algorithms to analyze and derive insights from large, complex, and multimodal datasets makes them a promising tool for advancing nutrition research [98]. Efforts to use AI to enhance the consistency and granularity of UPF classification, thereby improving the quality of UPF research, are already underway. For example, Menichetti and colleagues developed FoodProX, a machine-learning classifier that predicts NOVA classifications based on nutritional data from the FNDDS. They also introduced FPro, a continuous index that quantifies the degree of food processing, which importantly introduces the ability to differentiate foods within NOVA categories [99]. A number of other AI algorithms have been developed for the purpose of classifying UPFs. For instance, Hu and colleagues recently published on fine-tuned language models that predict NOVA food categories based on ingredient lists [100], and Elbassuoni et al. introduced a deep learning NOVA classifier that classifies foods from images [101]. These tools aid in advancing objective UPF classification, but, with the exception of FPro, do not provide a more granular characterization of food processing beyond the NOVA categories. With the growing body of AI-powered applications that are based on or promoting the use of NOVA categories, the in-depth, evidence-based validation is critical to justify the reliability and usability of such tools.

**Figure 3:**
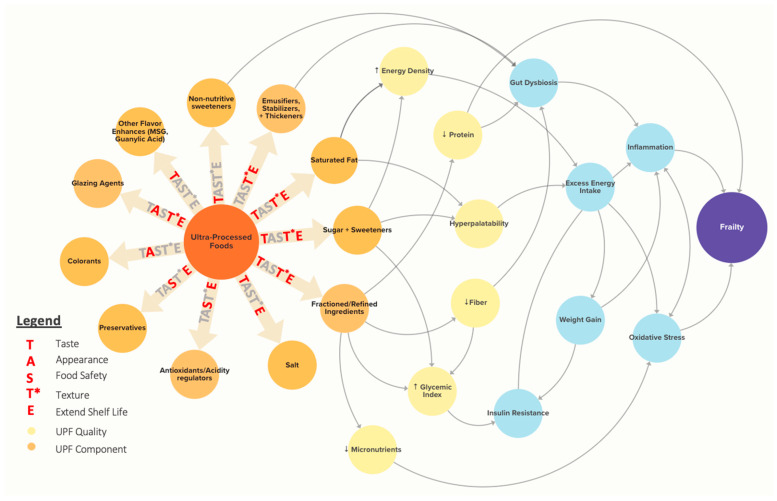
The TAST*E framework shows proposed causal mechanisms linking Ultra-Processed Foods (UPFs) to frailty. Common UPF contents (orange) and resulting UPF qualities (yellow) are linked to potential biological mechanisms (light blue) that are implicated in frailty. Importantly, the same UPF contents have utility in the food supply that should be considered in evaluating the UPF issue. These are highlighted with the TAST*E framework. The figure was created using Kumu Relationship Mapping Software (https://kumu.io/) [102].

Beyond improving classification, AI can also aid in the causal analysis of the relationship between UPFs and health and inform real-world recommendations on UPFs. Numerous characteristics of UPFs may contribute to health outcomes, including nutrient composition, sensory qualities, additives, and ingredients, through a range of unknown mechanisms, as shown in Figure 3. Rather than a single feature driving the association between UPF and poor health, it is likely that multiple interacting and sometimes contradictory effects contribute, with the exact permutation of contributing factors varying by food item.

Causal AI, a rapidly evolving field, aims to model, infer, and test causal relationships and is particularly relevant for exploring the cause-and-effect links between UPF intake and health outcomes. Unlike predictive algorithms, which rely on correlations and patterns between variables, causal AI seeks to identify true cause-and-effect relationships [103]. The fundamental process of causal AI closely mirrors the approach of epidemiologists: developing causal diagrams, analyzing observational data to infer causal relationships, and testing causal mechanisms [103]. Manual analysis, however, is constrained by the complexity and volume of potential relationships, whereas AI approaches are well-suited to navigate these issues. Causal Bayesian networks, for example, can navigate and identify relationships between all variables in a dataset, without pre-specified input on which variables might interact, enabling discovery in large, complex, and multimodal datasets [104]. Unlike predictive modeling, a causal AI approach reveals the exact relationships between variables and the intermediate mechanisms linking cause and effect (for example, the exact relationship between a food additive and mechanisms towards a health outcome) [104], which will be essential for targeted UPF interventions.

Beyond identifying the causal relationship between UPF intake and health outcomes, causal AI approaches can help policymakers understand the impact of potential recommendations or interventions on UPF intake while incorporating complex contextual factors. For example, in developing UPF reformulation policies, we must consider both the health effects of additives and ingredients in UPF foods as well as their functional roles in the food supply, including taste, appearance, food safety, texture, and extended shelf life (TAST*E in Figure 3). Using causal AI, we can explore “What-if?” questions for reformulation scenarios to assess not only the impact on consumer health but also potential effects on food properties and safety.

Thoughtful integration of AI with traditional epidemiological methods could help refine our understanding of UPFs and their health effects, ultimately guiding more precise and feasible dietary recommendations. Scientific expertise will be essential in overseeing and informing such efforts, particularly in weighing the strength of evidence that defines the relationships between food qualities, contents, and health. Key challenges remain, including data quality and harmonization, especially considering the limited availability of large databases with complete nutritional and ingredient information, and the scarcity of prospective cohort studies with such intensive dietary data.

## 5. Conclusions

The ultimate goal of UPF research is to determine how UPF intake causally affects human health, so that we can use this evidence to inform practical, evidence-based public health recommendations. The findings of our study contribute to the growing body of literature examining the relationship between UPF intake and chronic disease outcomes in aging. Our findings suggest UPF intake is related to modest declines in muscle function, but a relationship with frailty was not supported. We highlighted future research directions, including AI approaches, that have the potential to advance UPF research with greater granularity, nuance, and practical relevance but rely on access to high-quality datasets and continued expert input. Any food policies related to UPFs will need to consider not only the causal relationship between UPFs and health but also the potentially positive roles that UPFs play in ensuring food security in vulnerable populations like older adults and the integral role they currently play, for better or for worse, in our modern food system and ways of life.

## Figures and Tables

**Table 1:** Baseline characteristics of 2547 participants in the analytic sample and 431 participants excluded due to lack of frailty follow-up assessment in the Framingham Offspring Study.

Characteristics	Included Participants		Participants Excluded Due to Lack of Frailty Follow-Up Assessment	
	Mean (SD) or *n* (%)	*n*	Mean (SD) or *n* (%)	*n*
Follow-Up Time (years)	10.8 (2.7)	2547	-	-
Age (years)	60.3 (8.9)	2547	66.7 (9.6)	431
Female, *n* (%)	1402 (55.1)	2547	215 (49.9)	431
Education, *n* (%)		2547		431
Less than High School	87 (3.4)		18 (4.2)	
High School Graduate	708 (27.8)		144 (33.4)	
Some College	723 (28.4)		131 (30.4)	
College Graduate	990 (38.9)		113 (26.2)	
Missing	39 (1.5)		25 (5.8)	
Current Smoking, *n* (%)	283 (11.1)	2547	68 (15.8)	430
Health Status, *n* (%)		2547		430
Excellent	1231 (48.5)		127 (39.5)	
Good/Very Good	1187 (46.7)		260 (60.5)	
Fair/Poor	123 (4.8)		43 (10.0)	
UPF Intake (servings/day)	7.2 (2.9)	2547	7.3 (2.7)	431
Energy Intake (kcal/day)	1840.4 (594.3)	2547	1788.4 (587.8)	431
DASH Score	24.2 (5.2)	2547	23.7 (5.2)	431
Multivitamin Use, *n* (%)	1331 (52.3)	2545	220 (51.2)	430
BMI (kg/m^2^)	28.1 (5.3)	2547	28.5 (5.1)	431
Physical Activity Index	37.9 (6.2)	2510	37.8 (6.6)	418
Grip Strength (kg)	33.6 (12.9)	1905	30.4 (11.9)	290
Gait Speed (m/s)	1.3 (0.3)	2084	1.1 (0.3)	304
Exhaustion, *n* (%)	130 (5.1)	2533	28 (6.6)	427
Weight Loss, *n* (%)	47 (1.9)	2547	8 (1.9)	431
History of CVD, *n* (%)	257 (10.1)	2547	102 (23.7)	431
History of Cancer, *n* (%)	212 (8.3)	2547	69 (16.0)	431
History of Diabetes, *n* (%)	261 (10.3)	2547	89 (20.7)	431

**Table 2:** Association of Ultra-Processed Food (UPF) intake (energy-adjusted servings per day) and odds of developing frailty.

	Cumulative Logistic Regression		Mixed Logistic Regression	
	OR (95% CI)	*p* value	OR (95% CI)	*p* value
Model 1 ^a^	1.04 (0.98, 1.10)	0.16	0.97 (0.90, 1.04)	0.35
Model 2 ^b^	1.01 (0.95, 1.07)	0.81	0.98 (0.91, 1.10)	0.94
Model 3 ^c^	0.98 (0.92, 1.05)	0.55	0.93 (0.84, 1.02)	0.14

^a^ Model 1 is adjusted for baseline age, education, and sex; ^b^ Model 2 is adjusted for model 1 covariates and energy intake, multivitamin use, smoking, self-rated health score, history of diabetes, history of cancer, and history of cardiovascular disease; ^c^ Model 3 is adjusted for model 2 covariates and Dietary Approaches to Stop Hypertension score.

**Table 3:** Sex and age-stratified associations of UPF intake (energy-adjusted servings per day) and odds of developing frailty.

	Cumulative Logistic Regression		Mixed Logistic Regression	
	OR (95% CI) ^a^	P (Interaction) ^b^	OR (95% CI) ^a^	P (Interaction) ^b^
Sex				
Men (*n* = 1145)	0.95 (0.86, 1.06)	0.39	0.99 (0.81, 1.21)	0.70
Women (*n* = 1402)	1.07 (1.04, 1.09)		0.94 (0.83, 1.05)	
Baseline Age				
Baseline Age < 60 years (*n* = 1253)	1.05 (0.93, 1.20)	0.17	0.94 (0.78, 1.12)	0.68
Baseline Age ≥ 60 years (*n* = 1294)	0.95 (0.88, 1.03)		0.96 (0.85, 1.08)	

^a^ Models adjusted for baseline age, education, energy intake, multivitamin use, smoking, self-rated health score, history of diabetes, history of cancer, history of CVD, DASH score; ^b^ From likelihood ratio test comparing model fit with multiplicative interaction term to model without multiplicative interaction term.

**Table 4:** Association of Ultra-Processed Food intake (energy-adjusted servings per day) and annualized change in select frailty components.

	β (95% CI)	*p* Value
Annualized Change in Grip Strength, kg/year (*n* = 1872)		
Model 1 ^a^	−0.01 (−0.02, 0.00)	0.14
Model 2 ^b^	−0.01 (−0.02, 0.01)	0.23
Model 3 ^c^	−0.01 (−0.02, 0.01)	0.30
Annualized Change in Gait Speed, m/s/year (*n* = 2033)		
Model 1	−0.001 (−0.001, −0.002)	0.01 *
Model 2	−0.001 (−0.001, −0.0001)	0.03 *
Model 3	−0.001 (−0.001, −0.0001)	0.03 *
Annualized Change in Weight, lb/year (*n* = 2545)		
Model 1	−0.02 (−0.05, 0.00)	0.09
Model 2	−0.02 (−0.05, 0.01)	0.17
Model 3	−0.02 (−0.05, 0.01)	0.28

^a^ Model 1 is adjusted for baseline age, education, sex; ^b^ Model 2 is adjusted for model 1 covariates and energy intake, multivitamin use, smoking, self-rated health score, history of diabetes, history of cancer, and history of CVD, BMI, and physical activity index; ^c^ Model 3 is adjusted for model 2 covariates and DASH score; * *p* < 0.05.

**Table 5:** Sex- and age-stratified associations of UPF intake (energy-adjusted servings per day) and annualized change in select frailty components.

	β (95% CI) ^a^	*p* (Interaction) ^b^
Sex		
Annualized Change in Grip Strength, kg/year		
Men (*n* = 848)	−0.02 (−0.05, −0.001)	0.01 *
Women (*n* = 1024)	0.01 (−0.01, 0.03)	
Annualized Change in Gait Speed, m/s/year		
Men (*n* = 917)	−0.0004 (−0.001, 0.0004)	0.35
Women (*n* = 1116)	−0.001 (−0.002, −0.0003)	
Annualized Change in Weight, lb/year		
Men (*n* = 1145)	−0.02 (−0.06, 0.02)	0.98
Women (*n* = 1400)	−0.01 (−0.06, 0.03)	
Baseline Age		
Annualized Change in Grip Strength, kg/year		
<60 years (*n* = 959)	−0.02 (−0.04, −0.001)	0.14
≥60 years (*n* = 913)	0.01 (−0.01, 0.03)	
Annualized Change in Gait Speed, m/s/year		
<60 years (*n* = 1027)	−0.0004 (−0.001, 0.0002)	0.64
≥60 years (*n* = 1006)	−0.0008 (−0.002, 0.000)	
Annualized Change in Weight, lb/year		
<60 years (*n* = 1253)	−0.03 (−0.08, 0.01)	0.71
≥60 years (*n* = 1292)	0.001 (−0.04, 0.04)	

^a^ All models adjusted for baseline age, education, energy intake, multivitamin use, smoking, self-rated health score, history of diabetes, history of cancer, history of CVD, DASH score. Grip and Gait speed models are also adjusted for PAI score and BMI; ^b^ From ANOVA comparing model fit with multiplicative interaction term to model without multiplicative interaction term; * *p* < 0.05.

## Data Availability

Data used in this study were sourced from the BioLINCC repository (https://biolincc.nhlbi.nih.gov/home/).

## Supplementary Materials

The following supporting information can be downloaded at: https://www.mdpi.com/article/10.3390/nu17162631/s1, Table S1: Study Population Characteristics of 2547 Participants in the Analytic Sample by Low and High UPF Intake. Table S2: Sensitivity Analyses of the Association between UPF Intake (energy-adjusted servings per day) and Odds of Developing Frailty.

## References

[1] United Nations, Department of Economic and Social Affairs, Population Division (2017). World Population Ageing 2017—Highlights.

[2] Clegg A., Young J., Iliffe S., Rikkert M.O., Rockwood K. (2013). Frailty in elderly people. Lancet.

[3] Leng S., Chen X., Mao G. (2014). Frailty syndrome: An overview. Clin. Interv. Aging.

[4] Song X., Mitnitski A., Rockwood K. (2010). Prevalence and 10-Year Outcomes of Frailty in Older Adults in Relation to Deficit Accumulation. J. Am. Geriatr. Soc..

[5] Klein B.E.K., Klein R., Knudtson M.D., Lee K.E. (2005). Frailty, morbidity and survival. Arch. Gerontol. Geriatr..

[6] Kojima G. (2017). Frailty as a predictor of disabilities among community-dwelling older people: A systematic review and meta-analysis. Disabil. Rehabil..

[7] Fried L.P., Tangen C.M., Walston J., Newman A.B., Hirsch C., Gottdiener J., Seeman T., Tracy R., Kop W.J., Burke G. (2001). Frailty in Older Adults: Evidence for a Phenotype. J. Gerontol. A Biol. Sci. Med. Sci..

[8] Bandeen-Roche K., Seplaki C.L., Huang J., Buta B., Kalyani R.R., Varadhan R., Xue Q.-L., Walston J.D., Kasper J.D. (2015). Frailty in Older Adults: A Nationally Representative Profile in the United States. J. Gerontol. A Biol. Sci. Med. Sci..

[9] Young A.C.M., Glaser K., Spector T.D., Steves C.J. (2016). The Identification of Hereditary and Environmental Determinants of Frailty in a Cohort of UK Twins. Twin Res. Hum. Genet..

[10] Murabito J.M., Yuan R., Lunetta K.L. (2012). The Search for Longevity and Healthy Aging Genes: Insights From Epidemiological Studies and Samples of Long-Lived Individuals. J. Gerontol. A Biol. Sci. Med. Sci..

[11] Livshits G., Ni Lochlainn M., Malkin I., Bowyer R., Verdi S., Steves C.J., Williams F.M.K. (2018). Shared genetic influence on frailty and chronic widespread pain: A study from TwinsUK. Age Ageing.

[12] Dato S., Montesanto A., Lagani V., Jeune B., Christensen K., Passarino G. (2012). Frailty phenotypes in the elderly based on cluster analysis: A longitudinal study of two Danish cohorts. Evidence for a genetic influence on frailty. AGE.

[13] Zoico E., Roubenoff R. (2002). The Role of Cytokines in Regulating Protein Metabolism and Muscle Function. Nutr. Rev..

[14] Jeejeebhoy K.N. (2012). Malnutrition, fatigue, frailty, vulnerability, sarcopenia and cachexia: Overlap of clinical features. Curr. Opin. Clin. Nutr. Metab. Care.

[15] Fiatarone M.A., O’Neill E.F., Ryan N.D., Clements K.M., Solares G.R., Nelson M.E., Roberts S.B., Kehayias J.J., Lipsitz L.A., Evans W.J. (1994). Exercise Training and Nutritional Supplementation for Physical Frailty in Very Elderly People. N. Engl. J. Med..

[16] Semba R.D., Bartali B., Zhou J., Blaum C., Ko C.-W., Fried L.P. (2006). Low Serum Micronutrient Concentrations Predict Frailty Among Older Women Living in the Community. J. Gerontol. A Biol. Sci. Med. Sci..

[17] Rashidi Pour Fard N., Amirabdollahian F., Haghighatdoost F. (2019). Dietary patterns and frailty: A systematic review and meta-analysis. Nutr. Rev..

[18] Das A., Cumming R.G., Naganathan V., Blyth F., Ribeiro R.V., Le Couteur D.G., Handelsman D.J., Waite L.M., Simpson S.J., Hirani V. (2019). Prospective Associations Between Dietary Antioxidant Intake and Frailty in Older Australian Men: The Concord Health and Ageing in Men Project. J. Gerontol. Ser. A.

[19] Kobayashi S., Asakura K., Suga H., Sasaki S., The Three-Generation Study of Women on Diets and Health Study Groups (2014). Inverse association between dietary habits with high total antioxidant capacity and prevalence of frailty among elderly Japanese women: A multicenter cross-sectional study. J. Nutr. Health Aging.

[20] Millar C.L., Costa E., Jacques P.F., Dufour A.B., Kiel D.P., Hannan M.T., Sahni S. (2022). Adherence to the Mediterranean-style diet and high intake of total carotenoids reduces the odds of frailty over 11 years in older adults: Results from the Framingham Offspring Study. Am. J. Clin. Nutr..

[21] Kojima G., Avgerinou C., Iliffe S., Walters K. (2018). Adherence to Mediterranean Diet Reduces Incident Frailty Risk: Systematic Review and Meta-Analysis. J. Am. Geriatr. Soc..

[22] Ward R.E., Orkaby A.R., Chen J., Hshieh T.T., Driver J.A., Gaziano J.M., Djousse L. (2020). Association between Diet Quality and Frailty Prevalence in the Physicians’ Health Study. J. Am. Geriatr. Soc..

[23] Shivappa N., Stubbs B., Hébert J.R., Cesari M., Schofield P., Soysal P., Maggi S., Veronese N. (2018). The Relationship Between the Dietary Inflammatory Index and Incident Frailty: A Longitudinal Cohort Study. J. Am. Med. Dir. Assoc..

[24] Millar C.L., Dufour A.B., Shivappa N., Habtemariam D., Murabito J.M., Benjamin E.J., Hebert J.R., Kiel D.P., Hannan M.T., Sahni S. (2022). A proinflammatory diet is associated with increased odds of frailty after 12-year follow-up in a cohort of adults. Am. J. Clin. Nutr..

[25] Juul F., Parekh N., Martinez-Steele E., Monteiro C.A., Chang V.W. (2022). Ultra-processed food consumption among US adults from 2001 to 2018. Am. J. Clin. Nutr..

[26] Mariath A.B., Machado A.D., Ferreira L.D.N.M., Ribeiro S.M.L. (2022). The possible role of increased consumption of ultra-processed food products in the development of frailty: A threat for healthy ageing?. Br. J. Nutr..

[27] Monteiro C.A., Levy R.B., Claro R.M., De Castro I.R.R., Cannon G. (2010). Increasing consumption of ultra-processed foods and likely impact on human health: Evidence from Brazil. Public Health Nutr..

[28] Louzada M.L.D.C., Martins A.P.B., Canella D.S., Baraldi L.G., Levy R.B., Claro R.M., Moubarac J.-C., Cannon G., Monteiro C.A. (2015). Ultra-processed foods and the nutritional dietary profile in Brazil. Rev. Saúde Pública.

[29] Monteiro C.A., Cannon G., Lawrence M., da Costa Louzada M.L., Pereira Machado P. (2019). Ultra-Processed Foods, Diet Quality, and Health Using the NOVA Classification System.

[30] Hao J., Zhou P., Qiu H. (2022). Association between Ultra-Processed Food Consumption and Frailty in American Elder People: Evidence from a Cross-Sectional Study. J. Nutr. Health Aging.

[31] Zupo R., Donghia R., Castellana F., Bortone I., De Nucci S., Sila A., Tatoli R., Lampignano L., Sborgia G., Panza F. (2023). Ultra-processed food consumption and nutritional frailty in older age. GeroScience.

[32] Sandoval-Insausti H., Blanco-Rojo R., Graciani A., López-García E., Moreno-Franco B., Laclaustra M., Donat-Vargas C., Ordovás J.M., Rodríguez-Artalejo F., Guallar-Castillón P. (2020). Ultra-processed Food Consumption and Incident Frailty: A Prospective Cohort Study of Older Adults. J. Gerontol. Ser. A.

[33] Morley J.E., Malmstrom T.K., Miller D.K. (2012). A simple frailty questionnaire (FRAIL) predicts outcomes in middle aged African Americans. J. Nutr. Health Aging.

[34] Fung T.T., Rossato S.L., Chen Z., Khandpur N., Rodriguez-Artalejo F., Willett W.C., Struijk E.A., Lopez-Garcia E. (2024). Ultraprocessed foods, unprocessed or minimally processed foods, and risk of frailty in a cohort of United States females. Am. J. Clin. Nutr..

[35] Cordova R., Kliemann N., Huybrechts I., Rauber F., Vamos E.P., Levy R.B., Wagner K.-H., Viallon V., Casagrande C., Nicolas G. (2021). Consumption of ultra-processed foods associated with weight gain and obesity in adults: A multi-national cohort study. Clin. Nutr..

[36] Beslay M., Srour B., Méjean C., Allès B., Fiolet T., Debras C., Chazelas E., Deschasaux M., Wendeu-Foyet M.G., Hercberg S. (2020). Ultra-processed food intake in association with BMI change and risk of overweight and obesity: A prospective analysis of the French NutriNet-Santé cohort. PLoS Med..

[37] Mendonça R.D.D., Pimenta A.M., Gea A., De La Fuente-Arrillaga C., Martinez-Gonzalez M.A., Lopes A.C.S., Bes-Rastrollo M. (2016). Ultraprocessed food consumption and risk of overweight and obesity: The University of Navarra Follow-Up (SUN) cohort study. Am. J. Clin. Nutr..

[38] Canhada S.L., Luft V.C., Giatti L., Duncan B.B., Chor D., Fonseca M.D.J.M.D., Matos S.M.A., Molina M.D.C.B., Barreto S.M., Levy R.B. (2020). Ultra-processed foods, incident overweight and obesity, and longitudinal changes in weight and waist circumference: The Brazilian Longitudinal Study of Adult Health (ELSA-Brasil). Public Health Nutr..

[39] Willett W.C., Reynolds R.D., Cottrell-Hoehner S., Sampson L., Browne M.L. (1987). Validation of a semi-quantitative food frequency questionnaire: Comparison with a 1-year diet record. J. Am. Diet. Assoc..

[40] Rimm E.B., Giovannucci E.L., Stampfer M.J., Colditz G.A., Litin L.B., Willett W.C. (1992). Reproducibility and Validity of an Expanded Self-Administered Semiquantitative Food Frequency Questionnaire among Male Health Professionals. Am. J. Epidemiol..

[41] Khandpur N., Rossato S., Drouin-Chartier J.-P., Du M., Steele E.M., Sampson L., Monteiro C., Zhang F.F., Willett W., Fung T.T. (2021). Categorising ultra-processed foods in large-scale cohort studies: Evidence from the Nurses’ Health Studies, the Health Professionals Follow-up Study, and the Growing Up Today Study. J. Nutr. Sci..

[42] Willett W., Howe G., Kushi L. (1997). Adjustment for total energy intake in epidemiologic studies. Am. J. Clin. Nutr..

[43] Siefkas A.C., Millar C.L., Dufour A.B., Kiel D.P., Jacques P.F., Hannan M.T., Sahni S. (2023). Dairy Food Intake Is Not Associated With Frailty in Adults From the Framingham Heart Study. J. Acad. Nutr. Diet..

[44] Oei S., Millar C.L., Nguyen Lily T.N., Mukamal K.J., Kiel D.P., Lipsitz L.A., Hannan M.T., Sahni S. (2023). Higher intake of dietary flavonols, specifically dietary quercetin, is associated with lower odds of frailty onset over 12 years of follow-up among adults in the Framingham Heart Study. Am. J. Clin. Nutr..

[45] Sahni S., Jacques P., Dufour A., Millar C., Kiel D., Hannan M. (2020). Total Carotenoid Intake Reduces the Odds of Frailty over 9 Years in Older Adults: Results from the Framingham Offspring Study. Curr. Dev. Nutr..

[46] Millar C.L., Dufour A.B., Hebert J.R., Shivappa N., Okereke O.I., Kiel D.P., Hannan M.T., Sahni S. (2023). Association of Proinflammatory Diet With Frailty Onset Among Adults With and Without Depressive Symptoms: Results From the Framingham Offspring Study. J. Gerontol. Ser. A.

[47] Kannel W.B. (1979). Some Health Benefits of Physical Activity: The Framingham Study. Arch. Intern. Med..

[48] Ma J., Liu X., Zhang Y., Cheng H., Gao W., Lai C.-Q., Gabriel S., Gupta N., Vasan R.S., Levy D. (2022). Diet Quality Scores Are Positively Associated with Whole Blood–Derived Mitochondrial DNA Copy Number in the Framingham Heart Study. J. Nutr..

[49] Fung T.T. (2008). Adherence to a DASH-Style Diet and Risk of Coronary Heart Disease and Stroke in Women. Arch. Intern. Med..

[50] Vandenbroucke J.P., von Elm E., Altman D.G., Gøtzsche P.C., Mulrow C.D., Pocock S.J., Poole C., Schlesselman J.J., Egger M. (2007). STROBE Initiative Strengthening the Reporting of Observational Studies in Epidemiology (STROBE): Explanation and elaboration. PLoS Med..

[51] (1997). Relative validity and reproducibility of a diet history questionnaire in Spain. I. Foods. EPIC Group of Spain. European Prospective Investigation into Cancer and Nutrition. Int. J. Epidemiol..

[52] Braesco V., Souchon I., Sauvant P., Haurogné T., Maillot M., Féart C., Darmon N. (2022). Ultra-processed foods: How functional is the NOVA system?. Eur. J. Clin. Nutr..

[53] Mijnarends D.M., Schols J.M.G.A., Meijers J.M.M., Tan F.E.S., Verlaan S., Luiking Y.C., Morley J.E., Halfens R.J.G. (2015). Instruments to Assess Sarcopenia and Physical Frailty in Older People Living in a Community (Care) Setting: Similarities and Discrepancies. J. Am. Med. Dir. Assoc..

[54] Watanabe D., Kurotani K., Yoshida T., Nanri H., Watanabe Y., Date H., Itoi A., Goto C., Ishikawa-Takata K., Kimura M. (2022). Diet quality and physical or comprehensive frailty among older adults. Eur. J. Nutr..

[55] Struijk E.A., Hagan K.A., Fung T.T., Hu F.B., Rodríguez-Artalejo F., Lopez-Garcia E. (2020). Diet quality and risk of frailty among older women in the Nurses’ Health Study. Am. J. Clin. Nutr..

[56] Bollwein J., Diekmann R., Kaiser M.J., Bauer J.M., Uter W., Sieber C.C., Volkert D. (2013). Dietary Quality Is Related to Frailty in Community-Dwelling Older Adults. J. Gerontol. A Biol. Sci. Med. Sci..

[57] Shikany J.M., Barrett-Connor E., Ensrud K.E., Cawthon P.M., Lewis C.E., Dam T.-T.L., Shannon J., Redden D.T., for the Osteoporotic Fractures in Men (MrOS) Research Group (2014). Macronutrients, Diet Quality, and Frailty in Older Men. J. Gerontol. A Biol. Sci. Med. Sci..

[58] Parsons T.J., Papachristou E., Atkins J.L., Papacosta O., Ash S., Lennon L.T., Whincup P.H., Ramsay S.E., Wannamethee S.G. (2019). Physical frailty in older men: Prospective associations with diet quality and patterns. Age Ageing.

[59] Jayanama K., Theou O., Godin J., Cahill L., Shivappa N., Hébert J.R., Wirth M.D., Park Y.-M., Fung T.T., Rockwood K. (2021). Relationship between diet quality scores and the risk of frailty and mortality in adults across a wide age spectrum. BMC Med..

[60] Monteiro C.A., Cannon G., Levy R.B., Moubarac J.-C., Louzada M.L., Rauber F., Khandpur N., Cediel G., Neri D., Martinez-Steele E. (2019). Ultra-processed foods: What they are and how to identify them. Public Health Nutr..

[61] Shim J.-S., Shim S.Y., Cha H.-J., Kim J., Kim H.C. (2022). Association between Ultra-processed Food Consumption and Dietary Intake and Diet Quality in Korean Adults. J. Acad. Nutr. Diet..

[62] Calixto Andrade G., Julia C., Deschamps V., Srour B., Hercberg S., Kesse-Guyot E., Allès B., Chazelas E., Deschasaux M., Touvier M. (2021). Consumption of Ultra-Processed Food and Its Association with Sociodemographic Characteristics and Diet Quality in a Representative Sample of French Adults. Nutrients.

[63] Moubarac J.-C., Batal M., Louzada M.L., Martinez Steele E., Monteiro C.A. (2017). Consumption of ultra-processed foods predicts diet quality in Canada. Appetite.

[64] Liu J., Steele E.M., Li Y., Karageorgou D., Micha R., Monteiro C.A., Mozaffarian D. (2022). Consumption of Ultraprocessed Foods and Diet Quality Among U.S. Children and Adults. Am. J. Prev. Med..

[65] Lauria F., Dello Russo M., Formisano A., De Henauw S., Hebestreit A., Hunsberger M., Krogh V., Intemann T., Lissner L., Molnar D. (2021). Ultra-processed foods consumption and diet quality of European children, adolescents and adults: Results from the I.Family study. Nutr. Metab. Cardiovasc. Dis..

[66] Buchner D.M., Cress M.E., Esselman P.C., Margherita A.J., De Lateur B.J., Campbell A.J., Wagner E.H. (1996). Factors Associated With Changes in Gait Speed in Older Adults. J. Gerontol. A Biol. Sci. Med. Sci..

[67] Perera S., Patel K.V., Rosano C., Rubin S.M., Satterfield S., Harris T., Ensrud K., Orwoll E., Lee C.G., Chandler J.M. (2016). Gait Speed Predicts Incident Disability: A Pooled Analysis. J. Gerontol. A Biol. Sci. Med. Sci..

[68] Shinkai S. (2000). Walking speed as a good predictor for the onset of functional dependence in a Japanese rural community population. Age Ageing.

[69] Studenski S. (2011). Gait Speed and Survival in Older Adults. JAMA.

[70] Ahmed-Yousef N.S., Dilian O., Iktilat K., Agmon M. (2023). CRP, but not fibrinogen, is associated with gait speed as early as middle age, in females but not males. Sci. Rep..

[71] Baptista G., Dupuy A.-M., Jaussent A., Durant R., Ventura E., Sauguet P., Picot M.-C., Jeandel C., Cristol J.P. (2012). Low-grade chronic inflammation and superoxide anion production by NADPH oxidase are the main determinants of physical frailty in older adults. Free Radic. Res..

[72] Verghese J., Holtzer R., Oh-Park M., Derby C.A., Lipton R.B., Wang C. (2011). Inflammatory Markers and Gait Speed Decline in Older Adults. J. Gerontol. A Biol. Sci. Med. Sci..

[73] Tristan Asensi M., Napoletano A., Sofi F., Dinu M. (2023). Low-Grade Inflammation and Ultra-Processed Foods Consumption: A Review. Nutrients.

[74] Zhang S., Gu Y., Rayamajhi S., Thapa A., Meng G., Zhang Q., Liu L., Wu H., Zhang T., Wang X. (2022). Ultra-processed food intake is associated with grip strength decline in middle-aged and older adults: A prospective analysis of the TCLSIH study. Eur. J. Nutr..

[75] Payette H., Hanusaik N., Boutier V., Morais J., Gray-Donald K. (1998). Muscle strength and functional mobility in relation to lean body mass in free-living frail elderly women. Eur. J. Clin. Nutr..

[76] Schaap L.A., Van Schoor N.M., Lips P., Visser M. (2018). Associations of Sarcopenia Definitions, and Their Components, With the Incidence of Recurrent Falling and Fractures: The Longitudinal Aging Study Amsterdam. J. Gerontol. Ser. A.

[77] Cruz-Jentoft A.J., Bahat G., Bauer J., Boirie Y., Bruyère O., Cederholm T., Cooper C., Landi F., Rolland Y., Sayer A.A. (2019). Sarcopenia: Revised European consensus on definition and diagnosis. Age Ageing.

[78] Kong W., Xie Y., Hu J., Ding W., Cao C. (2024). Higher ultra processed foods intake is associated with low muscle mass in young to middle-aged adults: A cross-sectional NHANES study. Front. Nutr..

[79] Jung S., Seo J., Kim J.Y., Park S. (2024). Associations of Ultra-Processed Food Intake with Body Fat and Skeletal Muscle Mass by Sociodemographic Factors. Diabetes Metab. J..

[80] Sun W., Liu J., Steele E.M., Yang X., Gao R., Wang C., Liu J. (2024). Association of ultra-processed food consumption with muscle mass among young and middle-aged US adults. Eur. J. Nutr..

[81] Rudakoff L.C.S., Magalhães E.I.D.S., Viola P.C.D.A.F., De Oliveira B.R., Da Silva Coelho C.C.N., Bragança M.L.B.M., Arruda S.P.M., Cardoso V.C., Bettiol H., Barbieri M.A. (2022). Ultra-processed food consumption is associated with increase in fat mass and decrease in lean mass in Brazilian women: A cohort study. Front. Nutr..

[82] Wallace J.I., Schwartz R.S. (2002). Epidemiology of weight loss in humans with special reference to wasting in the elderly. Int. J. Cardiol..

[83] Chumlea W.C., Garry P.J., Hunt W.C., Rhyne R.L. (1988). Distributions of serial changes in stature and weight in a healthy elderly population. Hum. Biol..

[84] DiMilia P.R., Mittman A.C., Batsis J.A. (2019). Benefit-to-Risk Balance of Weight Loss Interventions in Older Adults with Obesity. Curr. Diab. Rep..

[85] McMinn J., Steel C., Bowman A. (2011). Investigation and management of unintentional weight loss in older adults. BMJ.

[86] Rothman M.D., Leo-Summers L., Gill T.M. (2008). Prognostic Significance of Potential Frailty Criteria. J. Am. Geriatr. Soc..

[87] Han T.S., Tajar A., Lean M.E.J. (2011). Obesity and weight management in the elderly. Br. Med. Bull..

[88] Leonberg K.E., Maski M.R., Scott T.M., Naumova E.N. (2025). Ultra-Processed Food and Chronic Kidney Disease Risk: A Systematic Review, Meta-Analysis, and Recommendations. Nutrients.

[89] Leonberg K.E., Maski M.R., Scott T.M., Chen Y., Zhou B., Naumova E.N. (2025). Trends in chronic kidney disease and calories from ultra-processed foods: NHANES at the highly granular level. Discov. Public Health.

[90] Huffman G.B. (2002). Evaluating and treating unintentional weight loss in the elderly. Am. Fam. Physician.

[91] Sahyoun N.R., Jacques P.F., Zhang X.L., Juan W., McKeown N.M. (2006). Whole-grain intake is inversely associated with the metabolic syndrome and mortality in older adults. Am. J. Clin. Nutr..

[92] McKeown N.M., Yoshida M., Shea M.K., Jacques P.F., Lichtenstein A.H., Rogers G., Booth S.L., Saltzman E. (2009). Whole-Grain Intake and Cereal Fiber Are Associated with Lower Abdominal Adiposity in Older Adults. J. Nutr..

[93] Maras J.E., Newby P.K., Bakun P.J., Ferrucci L., Tucker K.L. (2009). Whole grain intake: The Baltimore Longitudinal Study of Aging. J. Food Compos. Anal..

[94] Monge A., Silva Canella D., López-Olmedo N., Lajous M., Cortés-Valencia A., Stern D. (2021). Ultraprocessed beverages and processed meats increase the incidence of hypertension in Mexican women. Br. J. Nutr..

[95] Romieu I., Khandpur N., Katsikari A., Biessy C., Torres-Mejía G., Ángeles-Llerenas A., Alvarado-Cabrero I., Sánchez G.I., Maldonado M.E., Porras C. (2022). Consumption of industrial processed foods and risk of premenopausal breast cancer among Latin American women: The PRECAMA study. BMJ Nutr. Prev. Health.

[96] Dinu M., Bonaccio M., Martini D., Madarena M.P., Vitale M., Pagliai G., Esposito S., Ferraris C., Guglielmetti M., Rosi A. (2021). Reproducibility and validity of a food-frequency questionnaire (NFFQ) to assess food consumption based on the NOVA classification in adults. Int. J. Food Sci. Nutr..

[97] Ravandi B., Ispirova G., Sebek M., Mehler P., Barabási A.-L., Menichetti G. (2025). Prevalence of processed foods in major US grocery stores. Nat. Food.

[98] Côté M., Lamarche B. (2022). Artificial intelligence in nutrition research: Perspectives on current and future applications. Appl. Physiol. Nutr. Metab..

[99] Menichetti G., Ravandi B., Mozaffarian D., Barabási A.-L. (2023). Machine learning prediction of the degree of food processing. Nat. Commun..

[100] Hu G., Flexner N., Tiscornia M.V., L’Abbé M.R. (2023). Accelerating the Classification of NOVA Food Processing Levels Using a Fine-Tuned Language Model: A Multi-Country Study. Nutrients.

[101] Elbassuoni S., Ghattas H., Ati J.E., Shmayssani Z., Katerji S., Zoughbi Y., Semaan A., Akl C., Gharbia H.B., Sassi S. (2022). DeepNOVA: A Deep Learning NOVA Classifier for Food Images. IEEE Access.

[102] Kumu (2025). Relationship Mapping Software.

[103] Naumova E.N. (2024). Causal AI for public health research and policy: A journey back to the future. J. Public Health Policy.

[104] Sgaier S.K., Huang V., Charles G. (2020). The Case for Causal AI. Stanf. Soc. Innov. Rev..

