# Supplementary material for: Ultra-Processed Food and Frailty: Evidence from a Prospective Cohort Study and Implications for Future Research
Source: Nutrients. 2025 Aug 14;17(16):2631. doi: 10.3390/nu17162631 (PMC12389690; doi:10.3390/nu17162631)
Supplement: Supplementary file 1 [file nutrients-17-02631-s001.zip › nutrients-3779152-supplementary.pdf]

# Supplementary Materials

**Table S1:** Study Population Characteristics of 2,547 Participants in the Analytic Sample by Low and High UPF Intake

| | **Low UPF Intake (<6.7 servings per day)** | | **High UPF Intake (≥ 6.7 servings per day)** | |
|---|---|---|---|---|
| | **Mean (SD) or N (%)** | **n** | **Mean (SD) or N (%)** | **n** |
| Follow-Up Time (years) | 10.9 (2.7) | 1274 | 10.8 (2.7) | 1273 |
| Age (years) | 59.3 (8.9) | 1274 | 61.3 (9.0) | 1273 |
| Female, N (%) | 740 (58.1) | 1274 | 662 (52.0) | 1273 |
| Education, N (%) | | 1274 | | 1273 |
| Less than High School | 39 (3.1) | | 48 (3.8) | |
| High School Graduate | 309 (24.3) | | 399 (31.3) | |
| Some College | 375 (29.4) | | 348 (27.3) | |
| College Graduate | 530 (41.6) | | 460 (36.1) | |
| Missing | 21 (1.7) | | 18 (1.4) | |
| Current Smoking, N (%) | 141 (11.1) | 1274 | 142 (11.2) | 1273 |
| Health Status, N (%) | | 1272 | | 1269 |
| Excellent | 655 (51.5) | | 576 (45.4) | |
| Good/Very Good | 571 (44.9) | | 616 (48.5) | |
| Fair/Poor | 46 (3.6) | | 77 (6.1) | |
| UPF Intake (servings/day) | 5.1 (1.4) | 1274 | 9.3 (2.6) | 1273 |
| Energy Intake (kcal/day) | 1865.2 (591.0) | 1274 | 1815.5 (596.7) | 1273 |
| DASH Score | 25.5 (5.3) | 1274 | 22.8 (4.7) | 1273 |
| Multivitamin Use, N (%) | 698 (54.9) | 1272 | 663 (49.7) | 1273 |
| BMI ($kg/m^2$) | 27.7 (5.2) | 1274 | 28.5 (5.4) | 1273 |
| Physical Activity Index | 38.2 (6.3) | 1274 | 37.6 (6.1) | 1259 |
| Grip Strength (kg) | 33.6 (12.6) | 966 | 33.6 (13.1) | 939 |
| Gait Speed (m/s) | 1.3 (0.3) | 1045 | 1.2 (0.3) | 1039 |
| Exhaustion, N (%) | 71 (5.6) | 1268 | 59 (4.7) | 1265 |
| Weight Loss, N (%) | 28 (2.2) | 1274 | 19 (1.5) | 1273 |
| History of CVD, N (%) | 107 (8.4) | 1274 | 150 (11.8) | 1273 |
| History of Cancer, N (%) | 99 (7.8) | 1274 | 113 (8.9) | 1273 |
| History of Diabetes, N (%) | 106 (8.3) | 1274 | 155 (12.2) | 1273 |

**Table S2:** Sensitivity Analyses of the Association between UPF Intake (energy-adjusted servings per day) and Odds of Developing Frailty

| | Cumulative Logistic Regression | | Mixed Logistic Regression | |
|---|---|---|---|---|
| | OR (95 % CI) | *P* value | OR (95 % CI) | *P* value |
| *1) Frailty characterized without imputed BMI, weight, or height values.* | | | | |
| Model 1[a] | 1.02 (0.96, 1.09) | 0.46 | 0.98 (0.91, 1.05) | 0.52 |
| Model 2[b] | 1.08 (0.93, 1.06) | 0.77 | 1.00 (0.92, 1.10) | 0.93 |
| Model 3[c] | 1.08 (10.6, 1.10) | 0.24 | 0.94 (0.85, 1.03) | 0.18 |
| *2) Functional limitation for grip and gait coded as missing weakness or slowness criteria* | | | | |
| Model 1[a] | 1.04 (0.98, 1.10) | 0.24 | 0.97 (0.90, 1.04) | 0.43 |
| Model 2[b] | 1.00 (0.94, 1.07) | 0.94 | 1.00 (0.91, 1.10) | 0.99 |
| Model 3[c] | 0.97 (0.91, 1.04) | 0.44 | 0.95 (0.86, 1.04) | 0.26 |
| *3) UPF as cumulative average from examination 5* | | | | |
| Model 1[a] | 1.06 (0.98, 1.13) | 0.06 | 0.83 (0.71, 0.96) | 0.01* |
| Model 2[b] | 1.03 (0.96, 1.09) | 0.44 | 0.74 (0.57, 0.95) | 0.02* |
| Model 3[c] | 1.00 (0.94, 1.07) | 0.99 | 0.62 (0.46, 0.82) | <0.01* |

[a] Model 1 is adjusted for baseline age, education, sex [b] Model 2 is adjusted for model 1 covariates and energy intake, multivitamin use, smoking, self-rated health score, history of diabetes, history of cancer, and history of CVD [c] Model 3 is adjusted for model 2 covariates and DASH score
